# Familiarity enhances the effectiveness of odors as cues in paired-associate memory

**DOI:** 10.3758/s13421-025-01790-1

**Published:** 2025-09-03

**Authors:** Mohammad Hamzeloo, Luisa Bogenschütz, Ryan P. M. Hackländer, Christina Bermeitinger

**Affiliations:** Department of Psychology, University of Hildesheim, Universitätsplatz 1, 31141 Hildesheim, Germany

**Keywords:** Odor familiarity, Odor frequency, Odor discrimination, Odor training, Olfactory paired-association memory

## Abstract

Previous olfactory paired-associate (PA) studies showed that odors are less effective associative cues than other sensory modalities. It has been suggested that odor familiarity might improve memory stability, thereby facilitating PA memory performance. In the current study, we designed three experiments to investigate whether enhanced odor familiarity would also enhance the effectiveness of odors in a PA memory paradigm. In Experiments 1 and 2, subjects associated target shapes with eight familiar and eight unfamiliar odors (based on normative odor ratings in Experiment 1 and individual ratings in Experiment 2). The results of both experiments indicated that familiar odor cues were more effective in the retrieval of targets than unfamiliar odor cues. In Experiment 3, we investigated whether becoming (more) familiar with odors would also enhance the effectiveness of odors as cues in an olfactory PA test. An experimental group was familiarized with half of the pre-rated odors (16 odors, eight high and eight low familiar) over 4 weeks prior to the laboratory procedure while a control group was not. Both groups engaged in a PA memory paradigm in which they associated 32 odors (16 high and 16 low familiar odors) with black and white complex shapes. The results indicated that odor training led to enhanced PA learning for low-familiar odors in relation to untrained low-familiar odors. These results provide further evidence for odor familiarity as one of the critical features in olfactory processing and memory.

**Supplementary Information:**

The online version contains supplementary material available at 10.3758/s13421-025-01790-1.

## Introduction

Odors are often considered to be powerful triggers of past memories (Herz, 1998). Recent studies have reinforced this idea, showing that autobiographical memories triggered by odors are generally more emotional, pleasant, and vivid, yet less frequent, than those prompted by visual or verbal cues (for reviews, see Hackländer et al., 2019; Larsson et al., 2014). However, studies using a paired-associate (PA) paradigm to assess the ability to encode and retrieve associations to olfactory stimuli have found that odors are poorer cues than other stimuli, such as nonsense syllables (Bolger & Titchener, 1907; Heywood & Vortriede, 1905), abstract shapes (Davis, 1975, 1977), or auditory and verbal stimuli (Hamzeloo et al., 2025). This relative weakness of olfactory cues in PA memory highlights the need to examine the conditions, such as familiarity, that influence olfactory PA memory performance more closely.

In a typical olfactory PA paradigm, subjects are asked to associate an odor with a target stimulus (e.g., words, pictures, or shapes). At retrieval, the odor is presented as a cue to recall the target or to recognize it among other unpaired stimuli. This learning process involves the discrimination of odors, the discrimination of targets, and the formation of an association between them (Pennington & Rehder, 1995). It has been proposed that perceptual differences between olfaction and other sensory modalities or difficulties in odor discrimination and identification may explain the variation in PA performance (Schab, 1991).

Early research suggested that odors are processed as featureless stimuli, with minimal influence from semantic or verbal factors in episodic memory (Engen & Ross, 1973; Larjola & Von Wright, 1976; Lawless, 1978; Lawless & Cain, 1975; Lawless & Engen, 1977). However, more recent studies challenge this view, demonstrating that odor knowledge and experience are positively related to episodic odor memory performance (Larsson, 1997; Larsson & Bäckman, 1997; Rabin & Cain, 1984). For instance, Royet et al. (2013) showed that the ability to discriminate one odor from another and to identify an odor within a mixture improves with repeated odor exposure (Royet et al., 2013). Lyman and McDaniel (1986, 1990) found that odors that had been previously named or associated with a significant life episode were better remembered than odors that were visualized or simply smelled.

Perceived familiarity of an odor reflects prior experience with an odor, which may involve semantic knowledge if it includes general information about the odor, such as its typical context or associated category (Larsson, 1997). Odor familiarity is traditionally considered equivalent to frequency of previous stimulus exposure (Noble, 1953). Frequent exposure to an odor enhances a person’s potential ability to detect and/or discriminate, categorize, and, at the highest level, verbalize the odor (Wilson et al., 2009). Early studies demonstrated that odor familiarity significantly influences odor recognition (Herz & Engen, 1996). For instance, research by Engen and Ross (1973) showed that familiar odors are recognized more accurately and quickly than unfamiliar odors. Similarly, a study by Larsson et al. (2000) found that odors frequently encountered in daily life are more readily recognized. Recent research continues to support the idea that odor familiarity plays a crucial role in odor recognition. For example, a study by Kärnekull et al. (2015) demonstrated that odors familiar to individuals are recognized more accurately and rapidly than unfamiliar ones. These findings reinforce the notion that familiarity, often gained through repeated exposure but potentially influenced by other factors, is key to effective odor recognition.

Only two studies specifically evaluated the role of odor familiarity in PA memory (Davis, 1975, 1977). These studies demonstrated that participants were more successful in learning and recalling odor-digit pairs when the odors were familiar and dissimilar to others within the odor set, indicating that prior exposure enhances the associative learning process. Davis (1975) found that familiar odors serve as more effective cues in memory tasks, possibly due to deeper encoding. Besides the necessity of replicating the results of Davis and investigating PA memory with odor cues using new methods, the exact mechanisms by which familiarity or prior knowledge improves PA memory remain unknown, leaving open questions about how these processes enhance memory performance in such tasks.

To shed more light on the role of familiarity in odor PA memory, we conducted three experiments to evaluate how odor familiarity (pre-existing, individually rated, or obtained through training) improves odor PA memory performance. In Experiment 1, we selected high- and low-familiar odors from a common norm list with odor ratings (Moss et al., 2016). Consistent with previous research (Davis, 1975, 1977), we hypothesized that high-familiar odors would result in more accurate target recognition compared to low-familiar odors. In Experiment 2, we used high- and low-familiar odors as rated individually from each participant. Additionally, we aimed to explore different facets of odor familiarity, including direct odor familiarity rating, frequency of previous exposure, and discriminability. We hypothesized that odors rated as highly familiar, frequently encountered, and/or easily discriminable would lead to more accurate target recognition than those rated lower on these ratings. In Experiment 3, we did not only use odors that are already familiar to the participants, but we also used unfamiliar odors for which familiarity was increased during a training phase. We sought to determine whether increasing the familiarity of odors would enhance their effectiveness as cues in the PA memory task. We again hypothesized that familiar odors – even odors that become familiar through training – are more effective as potential cues in the PA memory paradigm.

## Experiment 1

The aim of this experiment was to explore the (facilitative) effect of odor familiarity in paired-associate (PA) memory involving odors. We used a common database with ratings on odors (Moss et al., 2016) for selecting high- and low-familiar odors. Experiment 1 served as a baseline for subsequent experiments. Given the positive correlation between odor familiarity and pleasantness (Engen & Ross, 1973; Lawless & Cain, 1975; Moss et al., 2016; Sulmont et al., 2002), we controlled for this variable by ensuring that familiar and unfamiliar odors had similar pleasantness ratings. We also refrained from including disgusting or highly unpleasant odors. We hypothesized that high-familiar odors would lead to more accurate and faster responses than low-familiar odors when used as cues for retrieving previously associated complex shapes.

### Method

#### Participants

We used G*Power v3.1.9.7 (Faul et al., 2007) to calculate the sample size for the experiment using a *t*-test to assess the difference between the two dependent means in a paired sample, a medium effect size of *d* =.5, and an α level of.05. The total sample size for statistical power of β =.80 was estimated to be *N* = 27. Considering the inclusion and exclusion criteria, we enrolled 30 volunteer students from the University of Hildesheim. General inclusion criteria were:

1. Normal or corrected-to-normal vision, hearing, and sense of smell;
2. No history of chronic or current disease;
3. No history of rhinitis, allergy, or asthma.

In addition, data were excluded from analyses if

1. The mean recognition accuracy was not significantly different from chance based on the result of a chi-squared test and a chance level of 25%;
2. There was a technical problem during the encoding or retrieval sessions.

To control for inclusion/exclusion criteria, participants underwent a standardized email or telephone screening before being invited to our study. In addition, each invited participant was instructed to refrain from using fragrant cosmetics and not to eat, smoke, or brush their teeth for at least 1 hour prior to the start of the experiment.

Three participants were excluded due to the average accuracy criterion, resulting in a final sample size of *N* = 27 (19 female, seven male, and one diverse). The mean age was 22.89 (*SD* = 4.31) years and the age range was 20−36 years.

#### Design

We implemented a one-factorial design with familiarity level (high vs. low) as the independent variable and response accuracy as well as reaction times (RTs) as the dependent measures.

### Material and apparatus

#### Odor stimuli

To select high- and low-familiar odors, we used normative data from a large norming study (Moss et al., 2016). In the first step, 16 high-familiar and 16 low-familiar odors were selected, such that the two groups did not differ significantly in pleasantness (see Table S1 in the Online Supplementary Materials for more information). Since the normative data were derived from a British sample, and to avoid any cultural differences in the rating of odor familiarity, we asked several colleagues, students, and research assistants to judge whether each of the 32 pre-selected odors was familiar to the German population. Based on these judgements, we then selected eight high-familiar (Marzipan, Sage, Baby powder, Vanilla, Soap Suds, Malted Barley, Out at Sea, and Aniseed Balls) and eight low-familiar (Sandalwood, Wood Chip, Earthy, Gingerbread, Leather/Hide, Cedar, Boiler room, and Peat) odors with high agreement to use in the PA paradigm. We also selected another eight odors (Buttered Popcorn, Pencils, Clove Oil, Cinder Toffee, Nag Champa, Hazelnut, Rockpools, and Candy Floss) with high disagreement on their familiarity to be used only in the rating phase. All the odors were supplied by AromaPrime company (https://aromaprime.com/) in liquid form.

#### Target stimuli

**Fig. 1:**
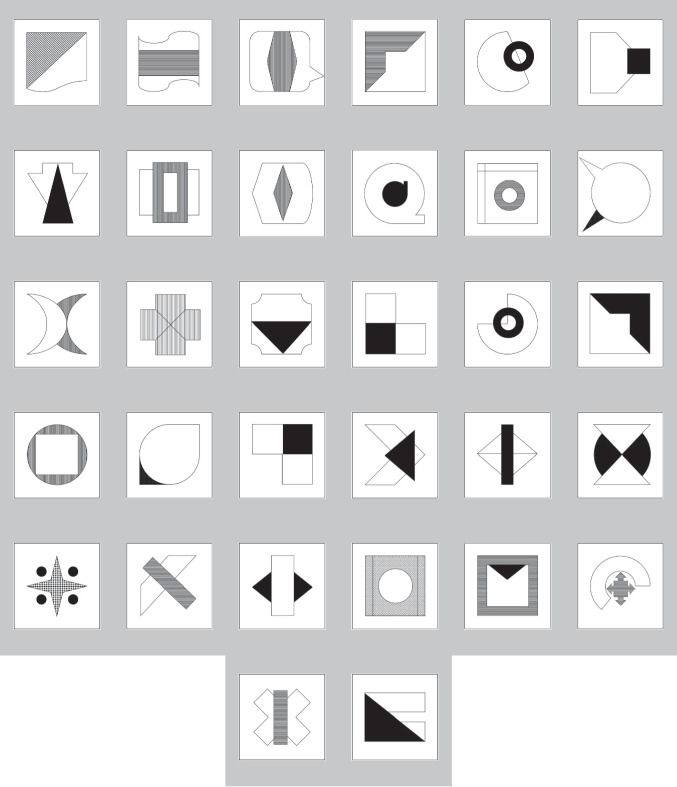
The target stimuli used in Experiments 1 and 2

Thirty-two black-and-white, complex, abstract shapes were used as targets in the PA paradigm. The stimuli were created by combining simple shapes with various patterns (see Fig. 1). The use of abstract shapes helped to ensure that there were no prior associations between the cues and the targets. The size of all shapes was 400 × 400 pixels and the vertical and horizontal resolution was 120 dpi.

#### Apparatus

The experimental task was programmed using E-Prime software (E-Prime 3.0, Psychology Software Tools, Pittsburgh, PA, USA) and administered on a Lenovo ThinkPad E550 laptop with a 15.6-in. display. All visual stimuli were presented in the center of the screen within a square frame with a white background inside on a gray background, and odors were presented by use of aroma stick pens (length: 10.02 cm, diameter: 0.77 cm, weight: 3 g) provided by Otto Hutt GmbH in Germany. Each stick contained 1.5 ml of aroma oil and was labeled with numbers (11−34) during the encoding phase and with letter codes (AB−AM, BA−BM) during the retrieval phase. The aroma stick pens were placed on two separate laboratory stick holders − one for encoding and another for retrieval − which were positioned on the table throughout the experiment.

#### Procedure

The study consisted of four phases: rating phase, encoding phase, distraction phase, and retrieval phase. At the beginning of the experiment, a research assistant ensured that the participants had followed the pre-study instructions. After signing the informed consent, participants were guided to the laboratory room equipped with a ventilator to prevent odor mixing. The ventilation system, which was centrally controlled, operated quietly during the experiment and did not produce any noticeable noise in the laboratory.

#### Rating phase

During the rating phase, participants were instructed to pick up the aroma stick corresponding to the number displayed on the screen, presented in a random order, and rate each odor on six dimensions: familiarity, frequency, pleasantness, irritability, context availability, and discriminability. Ratings were given using a 7-point Likert scale with labels at each end. Familiarity and frequency were assessed with three different questions each, and we averaged the responses to these three questions across participants to obtain overall familiarity and frequency scores. The other dimensions were assessed with a single question (see Table 1 for the English translations of the questions; the original German questions are provided in Table S2 in the Online Supplementary Materials). Additionally, age of acquisition and verbalizability were assessed through open-ended questions. For age of acquisition, participants specified the age (in years) when they first encountered the odor. For verbalizability, they were asked to provide any verbal labels they could associate with the odor, if possible.

**Table 1:** Questions for each rating in the rating phase

Question type	Lower scale	Upper scale
Familiarity		
How familiar is the smell to you?	Not at all	Very familiar
How familiar are you with this smell?	Not at all	Very familiar
How sure are you that you have smelled this smell before?	Not at all	Very sure
Frequency		
How often have you smelled this smell in your life?	Not at all	Very often
How often have you smelled this smell in the last 12 months?	Not at all	Very often
How often have you smelled this smell in the last 4 weeks?	Not at all	Very often
Pleasantness		
How pleasant do you find the smell?	Very unpleasant	Very pleasant
Irritability		
How arousing/exciting/activating do you find the smell?	Not at all	Very
Context availability		
How easily can you remember at least one situation in which you encountered the smell?	Not at all	Very
Discrimination		
How sure are you that you can distinguish this odor from other odors?	Not at all	Very sure
Age of acquisition		
At what age did you first notice this smell?	open-ended question	
Verbalizability		
What smell is that? If possible, please enter a verbal label for this smell.	open-ended question	

To rate an odor, participants had to pick up and open the aroma stick corresponding to the number presented on the screen. By pressing the spacebar, a sequence of rating questions appeared on the screen, and participants were asked to sniff the aroma stick and respond to each question in the presented order. The sequence of questions was the same for all odors and consistent across participants. They were allowed to sniff the stick as much as they needed to during the rating on all questions. To reset the sense of smell and mitigate olfactory fatigue, two 5-min breaks were included in the rating phase, one in the middle of the rating phase when they had completed the ratings for 12 aroma stick pens, and another at the end of the rating phase. During the breaks, participants were permitted to leave the experimental room and get fresh air.

#### Encoding phase

The encoding phase consisted of four learning blocks, each learning block consisting of eight training trials. During these trials, participants learned associations between the four stimulus pairs (i.e., the odor and the visual shape stimulus), with each pair presented twice in a random order. The training trials were followed by four practice trials. Both olfactory and visual stimuli were randomly distributed cross the learning blocks, and the order of the learning blocks was also randomized. This randomization was employed to control for potential differences in verbalizability and perceptibility of the visual stimuli that could influence task performance.

**Fig. 2:**
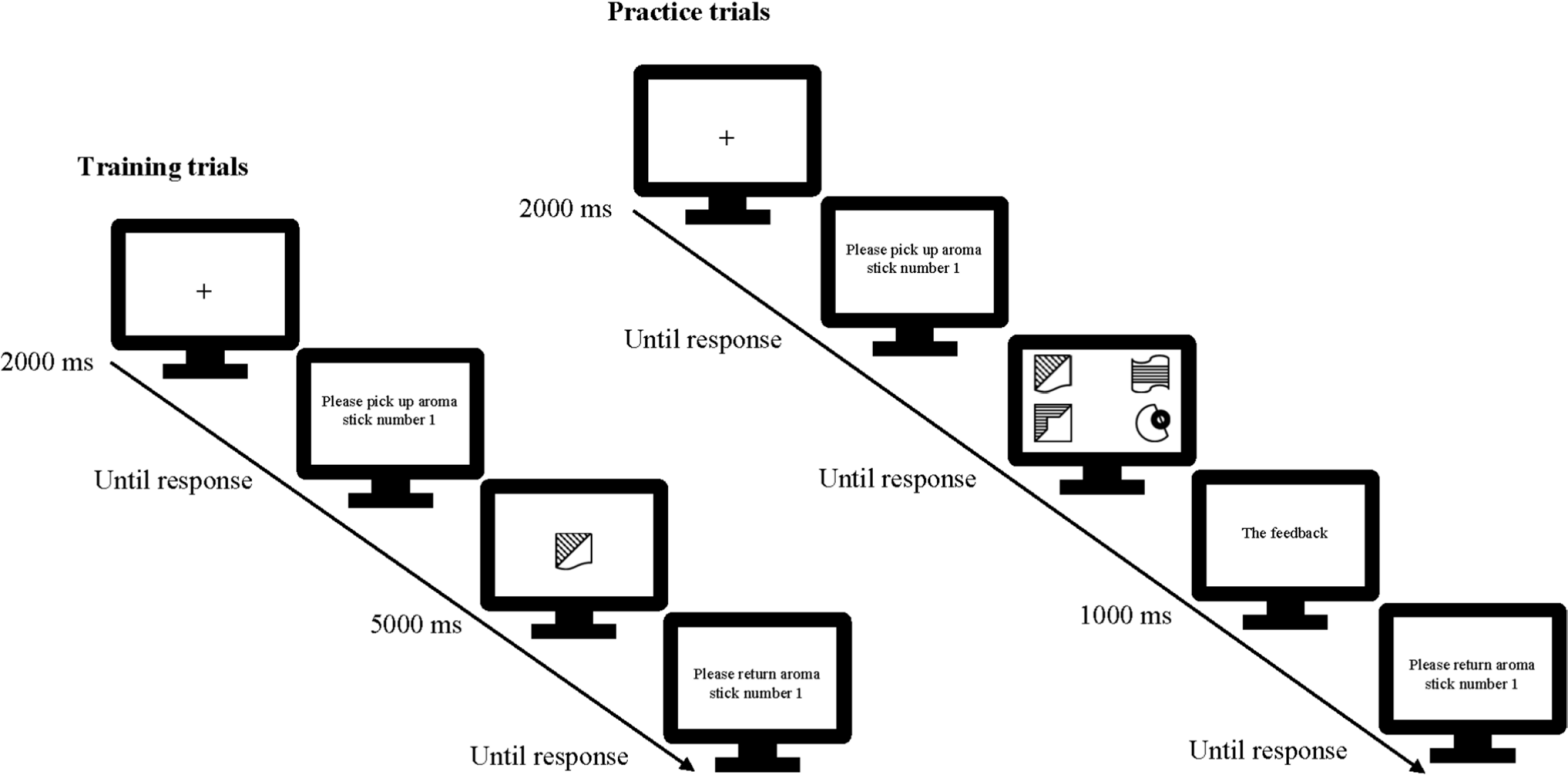
Procedure of a learning block in the encoding phase including training and practice trials in Experiment 1. Each learning block consisted of four training trials which were presented twice in the random order. There was no task for the participants other than to associate the two stimuli in each pair. There were four practice trials followed by training trials. The participants’ task was to click on the picture that was associated with the cue in the training trials. Incorrect trials were repeated at the end of the four practice trials in each block

As shown in Fig. 2, each training trial of a learning block began with a fixation cross in the center of the screen for 2,000 ms. Then, a message appeared on the screen asking participants to pick up an aroma stick from the holder in front of them. A number on the screen indicated which aroma stick should be picked up. Participants were instructed to open the aroma stick, press the spacebar, and start sniffing it. By pressing the spacebar, the target stimulus was presented in the center of the screen for the 5,000 ms. There was no task for the participants other than to associate between the odors and the complex shapes in each pair. After that, a message appeared on the screen asking the participant to return the aroma stick to its place.

After the training trials in each learning block there were four practice trials, which provided feedback concerning the participant’s accuracy on each trial. Each practice trial began with a fixation cross for 2,000 ms. After the fixation cross offset, a message appeared on the computer screen asking participants to pick up an aroma stick from the holder on the table. Again, a number on the screen indicated which aroma stick should be picked up. Participants had to press the spacebar when they were ready to continue. The task was to sniff the aroma stick and use it as a cue to identify the target shape among four images (of equal size and quality), one target and three foils from the other three pairs of the learning block, around the center of the screen, which remained until the participant responded by clicking on one of the shapes (see Fig. 2). The target shape was randomly assigned to one of the four possible positions around the center of the screen. After clicking on the shape, feedback (“correct” or “incorrect”) was presented in the middle of the screen for 1 s. Then a message appeared on the screen instructing the participant to return the aroma stick to its place. Incorrect trials were repeated at the end of the practice trials until participants gave a correct response. The order of the practice trials was also randomized.

This repetition protocol was designed to ensure that participants successfully encoded each odor-shape association before entering the test phase, thus minimizing the likelihood that test phase errors were due to encoding failure. Although the number of repetitions per item was not recorded due to a programming error, we conducted a follow-up analysis of response times during the training phase. This analysis examined whether any participants required considerably more time overall, which could indicate excessive repetitions. The mean response time across training trials was 2,681 ms (*SD* = 908.16), and no participant’s response time fell outside of ± 2 standard deviations from the mean. This suggests that no participant required substantially more training exposure than the others. No exclusion criteria or repetition limits were applied. All participants followed the same training structure, and we consider this procedure to be consistent across conditions.

#### Distraction phase

The distraction task consisted of 100 two-digit arithmetic addition equations (e.g., 26 + 46 = 52), and participants were instructed to calculate and judge whether the equation’s sum was true or false. All equations were presented in the center of the computer screen using Franklin Gothic Medium font, size 18 pt, after a fixation cross for 500 ms. They remained on the screen until participants provided a response to the trial. Participants’ responses were conveyed by clicking on the Yes or No button below the equation. The duration of the distraction phase was 5 min.

#### Retrieval phase

Upon termination of the distraction phase, the retrieval phase began, which consisted of 16 testing trials to evaluate the associations with 16 odors. The procedure for the testing trials was the same as for the practice trials in the encoding phase, except that no feedback was presented on participants’ responses and that letters instead of numbers appeared on the screen to indicate which stick should be picked-up.

#### Scoring protocol for odor ratings

Ratings including familiarity, frequency, pleasantness, irritability, context availability, and discrimination were rated on a scale of 1−7. For age of acquisition, when participants provided an age range as an answer, the mean value of that range was calculated. In the few cases where participants provided a qualitative answer to this question (e.g., “childhood”), it was not used in the analysis.

Scoring of odor labels for the verbalizability dimension was based on a modified version of the method used by Moss et al. (2016). Labels were scored on a 5-point scale (0−4): no response or a basic affective judgment scored 0, broad categorizations or generic labels (e.g., cleaner, food) scored 1, more specific group labels (e.g., floral, sweets) scored 2, any specific noun label scored 3, and the exact label matching the original odor scored 4. A score of 4 reflected both high specificity and correct identification, marking it as the upper bound of the verbalizability continuum. Two research assistants independently scored the labels, with the median score used as the final verbalizability score. The weighted Cohen’s kappa calculated *kw* = 0.61 (95% CI, 0.59−0.62), *p* <.0005), indicating moderate inter-rater agreement (McHugh, 2012).

#### Data analysis

Data were analyzed using SPSS (version 29), and graphs were generated using R (version 4.4.1) and the ggplot2 package (version 3.4.2, Wickham, 2016) for data visualization.

### Results

#### Ratings results

To compare our ratings with those of Moss et al., we averaged the ratings across participants for each odor on each scale. We found strong correlations between our ratings and those of Moss et al. for familiarity (*r* =.839, *p* <.001), frequency (*r* =.811, *p* <.001), context availability (*r* =.851, *p* <.001), discriminability (*r* =.884, *p* <.001), and verbalizability (*r* =.796, *p* <.001). Moderate correlations were observed for pleasantness (*r* =.535, *p* =.007), age of acquisition (*r* =.557, *p* =.005), and irritability (*r* =.498, *p* =.013). Additionally, our participants rated low familiar odors as less familiar than high familiar odors (*M*_low-familiar_ = 3.699, *M*_high-familiar_ = 5.018, *t*(22) = 5.092, *p* =.006). High familiar odors were also rated as slightly more pleasant than low familiar odors (*M*_low-familiar_ = 3.525, *M*_high-familiar_ = 4.247, *t*(22) = 2.12, *p* =.022).

#### Odor familiarity and PA memory performance

**Fig. 3:**
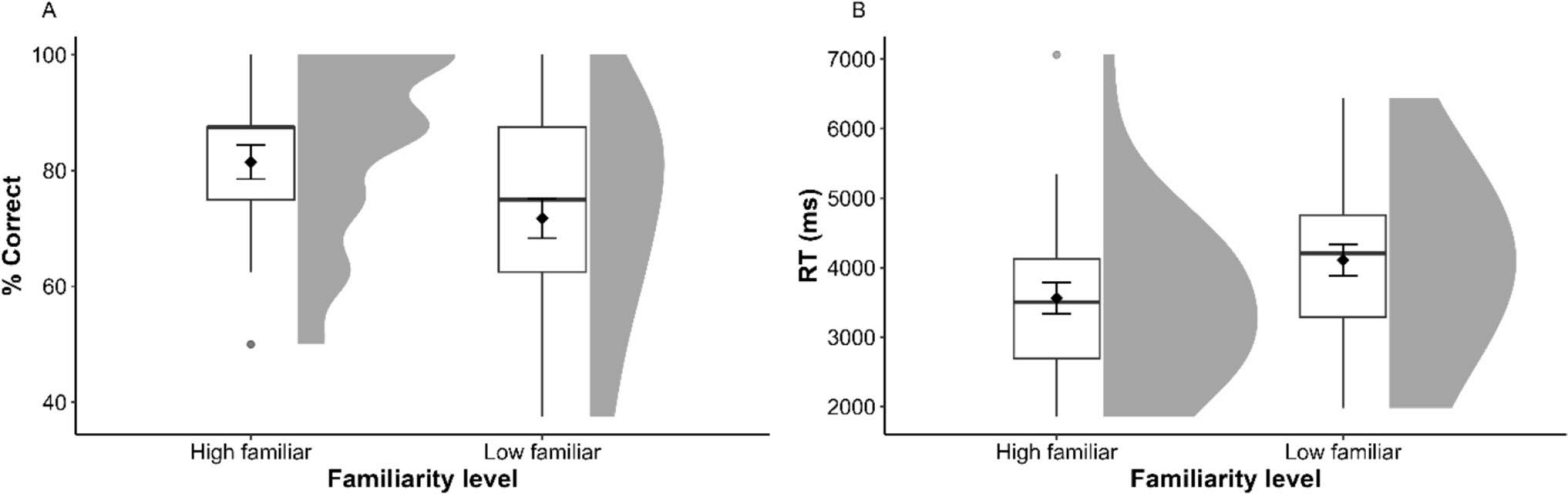
(**A**) Mean response accuracy and (**B**) reaction times (RTs) plotted separately for high- and low-familiar odors in the paired-associate memory paradigm in Experiment 1. The whiskers extend from the box to the smallest and largest values within 1.5 times the interquartile range from Q1 and Q3, respectively. Gray circles indicate values outside these hinges (outliers). The means (black diamond) and ±1 standard errors are superimposed on the boxplots. Half-violins plots show distribution density for each condition

To determine whether odor familiarity affects performance in odor PA memory, we performed two paired-sample *t*-tests on the two dependent variables: response accuracy and reaction time (RT) for high- and low-familiar odors. The results showed significant differences between the high and low-familiar odors in mean response accuracy (*t*(26) = 2.69, *p* =.006, Cohen’s* d* = 0.518) (Fig. 3A) and RTs (*t*(26) = 4.03, *p* <.001, Cohen’s* d* = 0.775) (Fig. 3B). These results confirmed our hypothesis, suggesting that high-familiar odors elicited more accurate and faster responses than low-familiar odors in the odor PA memory paradigm.^1^

### Discussion

This experiment highlights the impact of odor familiarity in odor PA memory performance by replicating previous findings (Davis, 1975, 1977) showing that familiarity enhances PA performance, leading to more accurate and faster responses. It has been suggested that odor familiarity facilitates odor associative memory by enhancing the encoding and the retrieval processes involved in memory formation (Herz, 1997; Larsson & Bäckman, 1993; Royet et al., 2013). These studies showed that familiar odors are more easily recognized and linked with associated stimuli, such as words or images, because they are encoded more deeply due to repeated exposure. This deep encoding creates stronger memory traces, making it easier to recall the associated information when prompted by the familiar odor. Additionally, familiar odors often evoke personal experiences and emotional responses, which further strengthen the associative memory process. The review by Herz and Engen (1996) supports this, showing that familiar odors are more effective cues in memory tasks, leading to better recall and more accurate associations compared to unfamiliar odors.

## Experiment 2

Odor familiarity is a complex construct influenced by various factors, such as frequency of prior exposure, age of acquisition, discriminability, and the descriptiveness of the odor. In Experiment 1, we selected high- and low-familiar odors from normative data (Moss et al., 2016) where perceived familiarity was assessed by asking participants in the norm study, “How familiar is this odor (not at all familiar/very familiar)?” Although Brown and Watson (1987) discussed that objective frequency measures may not reliably substitute for subjective familiarity ratings, earlier studies often equated frequency with familiarity. Recent research has found a strong correlation between odor familiarity rating, frequency, discriminability, and age of acquisition (Herz, 2000; Larsson & Bäckman, 1997; Moss et al., 2016) indicating that these factors may represent different aspects of the same underlying construct of prior experience with the odor.

Individual differences, such as variations in olfactory sensitivity, cognitive abilities, and prior experiences, can significantly influence how familiar an odor is perceived as being, thereby affecting both odor recognition and memory performance (Larsson et al., 2000). Therefore, we designed Experiment 2 to select high- and low-familiar odors based on participants’ ratings of the odors in three separate groups, as a between-subjects factor. We used either the direct rating of familiarity (odor familiarity direct rating group), or the frequency of odor exposure (odor frequency group), or the discriminability of odors (odor discrimination group) to select high versus low-familiar odors. We hypothesized that there should be no significant difference in PA memory performance of the three groups (direct odor familiarity rating group, odor frequency, and odor discrimination), as these measures all contribute to the broader concept of odor familiarity. Familiarity itself can be influenced by how often an odor is encountered and how easily it can be distinguished from other odors. We also hypothesized that high-familiar, frequent, and easily discriminable odors would lead to more accurate target recognition than those rated lower on these measures.

### Method

#### Participants

We initially recruited 99 naïve participants for this experiment, with the same inclusion and exclusion criteria as in Experiment 1. Ten students were excluded from the analysis due to the accuracy criterion (their overall accuracy in the PA paradigm was not significantly different from chance). A sensitivity analysis using G*Power v3.1.9.7 (Erdfelder et al., 2009) determined that the smallest effect we could detect with high probability given the final sample size (*N* = 89), a within-between interaction in an ANOVA, an α level of.05, and statistical power of *β* =.95 for three groups and two different measurements was *f* =.21. The final sample for the direct odor familiarity rating group was *N* = 29 (*M*_age_ = 21.93, *SD* = 1.91 years; two male); for the odor frequency group was *N* = 29 (*M*_age_ = 21.72, *SD* = 2.90 years; one male); and for the odor discrimination group was *N* = 31 (*M*_age_ = 22.90, *SD* = 3.87 years; eight male, and one diverse).

#### Design

We implemented a 2 × 3 factorial design with Familiarity Level (high vs. low) as the within-subject and Rating Groups (direct odor familiarity rating group, odor frequency, and odor discrimination) as the between-subject factors and response accuracy as well as RTs as the dependent measures.

#### Stimuli and procedure

All stimuli and the procedures used in Experiment 2 were identical to those used in Experiment 1, with one exception: the selection of low- and high-familiar odors used in the encoding and retrieval phases. Odors were selected based on the individual ratings on three different questions, each of which could evaluate one aspect of odor familiarity: odor familiarity, frequency, and discrimination. This was considered as a between-subjects factor and we recruited three groups of participants. For the first type of question, participants were asked to directly rate the familiarity of odors from “not at all” to “very familiar” in response to the typical question “How familiar is this odor to you?” (see Table 1). This was referred to as the *odor familiarity direct rating group*. In the second type of question, participants were asked to rate the odors from “not at all” to “very often” in response to the question “How often have you smelled this odor in your life?” This was referred to as the *odor frequency group*. In the third group, low- and high-familiar odors were selected based on their ratings from “not at all” to “very sure” in response to the question “How sure can you discriminate this odor from others?” This was referred to as the *odor discrimination group*.

To select high- and low-familiar odors for the encoding and retrieval phases, the familiarity rating scores for the corresponding question (24 odors rated on a scale from 1 to 7) were first sorted in ascending order for each participant individually. Lower ranks represented less familiar odors, while higher ranks indicated more familiar odors. Odors ranked below the eighth position were categorized as low-familiar, and those ranked above the 17th position were categorized as high-familiar. If an odor’s rating score matched the eighth or 17th cutoff, it was placed in a random pool. From this pool, odors were randomly selected until eight high- and eight low-familiar odors were assigned to each block of the encoding and retrieval phases. This process was implemented via the experimental program separately for each participant, ensuring that categorization was based on individual ratings rather than averaged or normative data.

### Results

To determine whether direct familiarity rating, frequency, and discrimination affect performance on odor PA memory, we performed two mixed ANOVAs on the two dependent variables: response accuracy and RT. The within-subject factor was Familiarity Level (high vs. low familiarity). We entered Rating Group (direct odor familiarity rating, odor frequency, and odor discriminability) as a between-subjects factor in the analyses. The results of the ANOVA on accuracy showed that the main effect of Familiarity Level was significant (*F*(1, 86) = 25.79, *p* <.001, ${\eta }_{p}^{2}$ =.231), but neither the main effect of the between-subject factor (Rating Group) (*F*(2, 86) =.48, *p* =.621, ${\eta }_{p}^{2}$ =.011) nor the interaction of Familiarity Level × Rating Group (*F*(2, 86) =.59, *p* =.557, ${\eta }_{p}^{2}$ =.014) was significant. Pairwise analyses with Bonferroni corrections showed that the accuracy of responses was higher for high-familiar than low-familiar odors in all three groups (direct odor familiarity rating: *M*_Difference_ = 10.672, *p* =.008, Cohen’s* d* = 0.551, odor frequency: *M*_Difference_ = 14.820, *p* <.001, Cohen’s* d* = 0.582, and odor discrimination: *M*_Difference_ = 8.975, *p* =.021, Cohen’s* d* = 0.481).

**Fig. 4:**
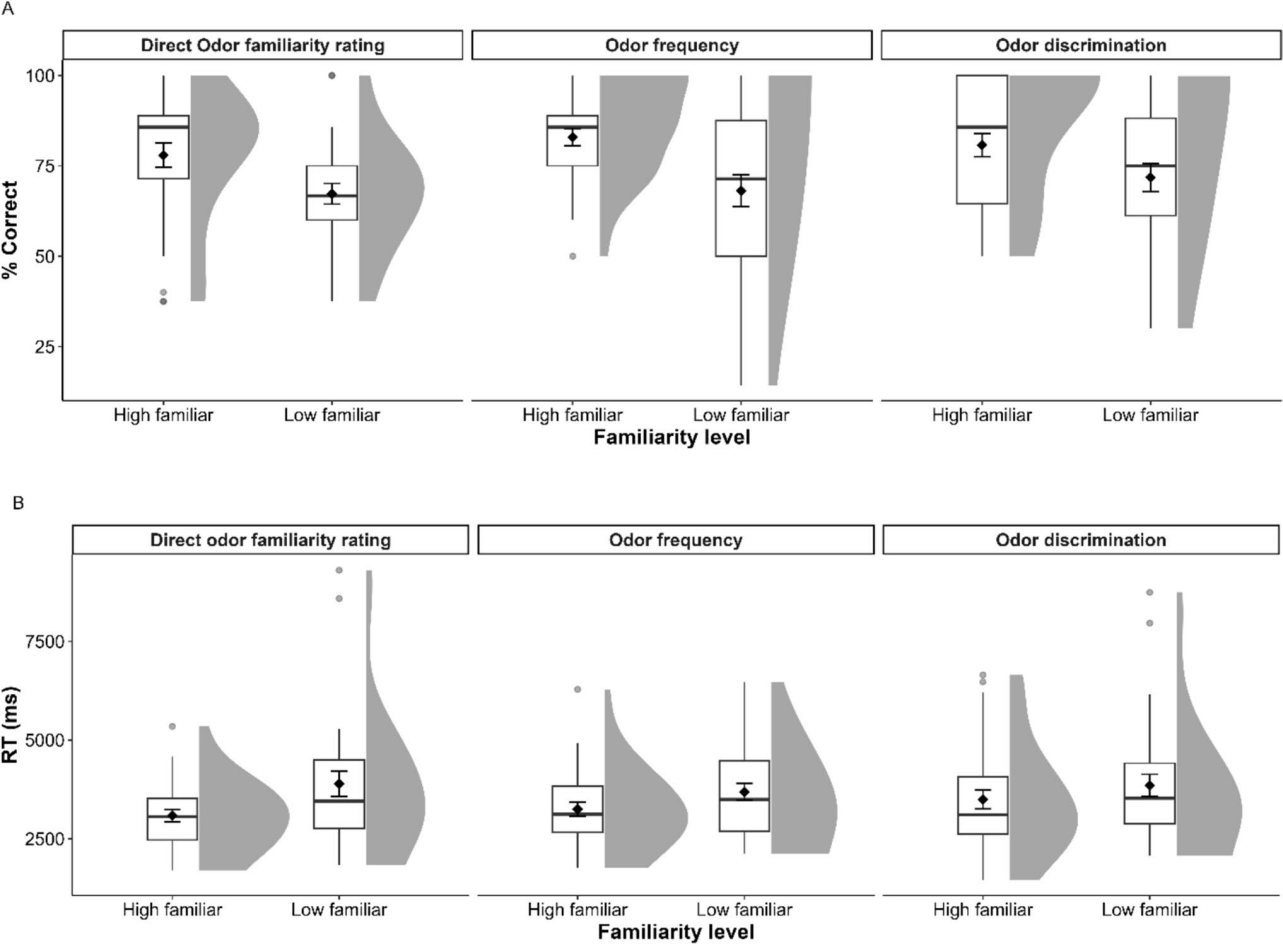
(**A**) Mean response accuracy and (**B**) reaction times (RTs) plotted separately for high- and low-familiar odors in the three groups of Experiment 2. The whiskers extend from the box to the smallest and largest values within 1.5 times the interquartile range from Q1 and Q3, respectively. Gray circles indicate values outside these hinges (outliers). The means (black diamond) and ±1 standard errors are superimposed on the boxplots. Half-violins plots show distribution density for each condition

The results of the mixed ANOVA on RTs showed the same pattern. The main effect of Familiarity Level was significant (*F*(1, 86) = 16.90, *p* <.001, ${\eta }_{p}^{2}$ =.164), but neither the main effect of the between-subjects factor (Rating Group) (*F*(2, 86) =.28, *p* =.753, ${\eta }_{p}^{2}$ =.007) nor the interaction of Familiarity Level × Rating Group (*F*(2, 86) = 1.14, *p* =.326, ${\eta }_{p}^{2}$ =.026) was significant. Pairwise analyses with Bonferroni corrections on RTs showed that RTs were faster for high-familiar than low-familiar odors in the odor familiarity group (*M*_Difference_ = 803 ms, *p* <.001, Cohen’s* d* = 0.597) and odor frequency (*M*_Difference_ = 436 ms, *p* =.047, Cohen’s* d* = 0.496) groups. Although high-familiar odors also elicited faster target recognition than low-familiar odors in the odor discrimination group, the difference was not significant (*M*_Difference_ = 354 ms, *p* =.109, Cohen’s* d* = 0.261) (see Fig. 4).

As an exploratory analysis, we examined whether familiarity ratings for each odor were related to paired-associate performance and reaction time. Correlating accuracy with familiarity ratings across participants for each odor yielded significant positive relationships in all three groups: odor discrimination (*r* =.583, *p* =.001), odor familiarity (*r* =.567, *p* =.002), and odor frequency (*r* =.481, *p* =.009). RTs showed a similar pattern, with significant positive correlations indicating faster responses for more familiar odors in the odor discrimination (*r* =.462, *p* =.012), odor familiarity (*r* =.403, *p* =.026), and odor frequency (*r* =.435, *p* =.015) groups. These findings are consistent with the main categorical analysis and support a link between odor familiarity and memory performance.

### Discussion

The above results of analyses with PA performance demonstrate that high-familiar, frequent odors facilitate PA performance by eliciting more accurate and faster target recognition. While easily discriminable odors also contributed to improved accuracy, our findings indicate that discriminability did not significantly influence RT, suggesting that its effect on faster target recognition may be limited. Notably, our findings replicate those of Experiment 1 and Davis (1975, 1977), further supporting the robustness of the odor familiarity effect on PA memory performance. Furthermore, this suggests that using standardized norm lists of rated odors from a separate sample (Exp. 1) or relying on individual ratings collected from the same participants who subsequently completed the memory test (Exp. 2) yielded the same results in assessing odor-related features that may influence odor memory performance.

Moreover, this finding suggests that different questions evaluating odor familiarity, frequency, and discriminability may be measuring a single construct, each of which taps into different aspects of prior knowledge about odors. Familiarity itself can be influenced by how often an odor is encountered (frequency), how easily it can be distinguished from other odors (discriminability), and how readily it can be identified or described. Each of these questions contributes to the overall sense of familiarity, thereby enhancing the cognitive processes involved in odor memory and recognition, particularly in terms of accuracy. While our results suggest that discriminability supports improved accuracy, its influence on RT was not significant, indicating that its role in facilitating faster recognition may be limited. These results are consistent with previous research showing that frequent exposure to an odor strengthens the neural pathways associated with that odor, making it easier to retrieve from memory (Rabin, 1988; Wilson & Stevenson, 2003). Taken together, these findings support the idea that familiarity with an odor is deeply rooted in prior experience, making it a powerful factor in improving PA performance and overall memory accuracy.

## Experiment 3

Experiments 1 and 2 demonstrated that high-familiar odors (whether determined by normative data in Experiment 1 or individually rated in Experiment 2) enhance performance on an odor PA memory task, eliciting higher accuracy and faster RTs. However, a challenge in using naturally familiar odors is that they come with participants’ pre-existing knowledge and experiences, which vary widely in frequency, context, and emotional or semantic associations. This experiential familiarity introduces variability that can influence PA memory performance, as prior encounters with odors may differ across individuals, confounding experimental control. In contrast, olfactory training offers a controlled method to manipulate familiarity by systematically exposing participants to specific odors in a structured, task-oriented setting, such as rating tasks over weeks. Controlled training differs from experiential familiarity in its precision and focus, potentially producing distinct effects on associative memory.

Structured olfactory training protocols, as used in clinical contexts, have been shown to improve olfactory function in patients with olfactory disorders (for more details, see these two recent systematic reviews: Pieniak et al., 2022; Vance et al., 2023). Other research using repeated odor exposure in healthy individuals (Dalton et al., 2002; Livermore & Hummel, 2004; Rabin & Cain, 1986) suggested that increased odor experience can also enhance olfactory sensitivity, discrimination, and identification, even within mixtures (Morquecho-Campos et al., 2019; Poupon et al., 2018). For instance, Jehl et al. (1995) demonstrated that familiarization with odor pairs before discrimination judgments reduced false alarms, particularly for dissimilar odor pairs, indicating benefits from repeated exposure. Moreover, previous research has shown that the benefits of olfactory training are not necessarily limited to the specific odors used during training. For instance, Morquecho-Campos et al. (2019) demonstrated that the training effects could transfer to novel odors or tasks, while Olofsson et al. (2020) showed that improvements extend even to an untrained visual memory task. More broadly, studies have found that olfactory training can enhance general olfactory function, including odor threshold, discrimination, and identification, even when the training involves only a limited set of odors (Damm et al., 2014; Hummel et al., 2009; Sorokowska et al., 2017). These findings suggest that olfactory training may engage domain-general mechanisms, allowing for transfer to untrained odors or broader olfactory functions.

Nevertheless, a key methodological limitation in parts of this literature is the absence of control groups (passive control or active control that engage in similar sniffing routines using unscented stimuli). Including such controls would allow a more rigorous assessment of whether training effects are truly driven by odor-specific mechanisms. Addressing these issues would help clarify what aspects of olfactory training are effective, and under what conditions. Taken together, it appears that manipulating odor familiarity in a controlled training setting potentially overcomes the limitations of using stimuli with pre-existing familiarity in experiments and allows us to assess its impact on PA memory performance.

The duration of the effects of olfactory training remains unclear, but studies have shown that improvements can emerge after only 3 weeks of training (Wang et al., 2004), within 1 week (Livermore & Hummel, 2004), within 5 days (Poupon et al., 2018), or even after repeating a test three times in a row (Rabin & Cain, 1986). These results indicate that a short-term olfactory training is sufficient to improve olfactory functions. By systematically exposing participants to specific odors, we can experimentally manipulate odor familiarity and investigate whether odor training improves the ability to form and retrieve associations. This approach not only provides insights into optimizing PA memory performance but also addresses the challenge of using stimuli that participants may already be familiar with, thereby enabling a more controlled exploration of the effects of odor familiarity on memory.

The current study aimed to explore whether a structured odor training phase designed to increase odor familiarity enhances the efficacy of odors in a PA memory paradigm. The control group, which did not undergo odor training phase, was included to serve as a baseline, allowing us to investigate whether mere odor exposure leads to general enhancement in olfactory PA memory or whether training benefits are specific to the odors trained during the odor training phase.

The familiarity levels (high vs. low) were selected to test our hypothesis that odor training would enhance the efficacy of low-familiar odors in the PA paradigm, as these odors are less likely to benefit from pre-existing knowledge, while high-familiar odors, already well known, may show minimal improvement. By including both trained and untrained odors, we assessed whether training effects were specific to the odors used in the training phase or if they transferred to untrained odors, addressing the specificity of the training effect. In addition, we examined how odor training influenced the eight rated odor characteristics (familiarity, frequency, pleasantness, irritability, context availability, discrimination, intensity, and complexity) to gain a comprehensive understanding of training-induced changes in odor perception.

This design was carefully structured to systematically test three hypotheses:

1. Odor training would change odor characteristics from the first to fourth training week;
2. 4 weeks of odor training, conducted once a week, would enhance PA memory performance for low-familiar odors, while high-familiar odors would show little to no improvement due to their pre-existing familiarity; and
3. Training effects would be specific to trained odors, with no transfer to untrained odors.

### Method

#### Participants

We initially recruited 75 naïve participants for this experiment (50 in the experimental group and 25 in the control group). The inclusion and exclusion criteria were the same as in Experiments 1 and 2. We chose to recruit twice as many participants for the experimental group because we had two different lists of odors for the training (see the *Stimuli* section for more details), requiring a larger sample size to ensure adequate data for each list. Participants in the experimental group had to complete all 4 weeks of odor training to be included in the analyses. They were excluded if they spent less than 10 min training in more than 1 week.

Fourteen participants were excluded from the experimental group: ten did not complete the training phase, and four did not spend the requisite time in the training sessions. Four participants in the control group were excluded from the analysis due to the accuracy criterion (their overall accuracy in the PA paradigm was not significantly different from chance). The final sample for the experimental group was *N* = 36 (*M*_age_ = 22.50, *SD* = 3.21 years; eight males) and for the control group was *N* = 21 (*M*_age_ = 22.33, *SD* = 2.89 years; 13 males). A sensitivity analysis using G*Power v3.1.9.7 (Erdfelder et al., 2009) determined that the smallest effect we could detect with high probability given the final sample size (*N* = 57), a within-between interaction in an ANOVA, an α level of.05, and statistical power of β =.95 for two groups and four different measurements, was *f* =.196.

#### Design

The experimental group completed a 2 × 2 within-subject design with the factors Familiarity Level (high vs. low) and Training Type (trained vs. untrained). The control group completed a single-factor within-subject design with Familiarity Level (high vs. low) only, as they did not undergo the odor training phase. Group (experimental vs. control) was included as a between-subject factor for planned comparisons. The dependent measures were response accuracy and RTs.

#### Stimuli

In this experiment, we selected 16 high-familiar and 16 low-familiar odors. Half of the odors (eight high-familiar and eight low-familiar) were selected based on participants’ ratings of the odors in Experiments 1 and 2. The other half were selected from the normative data provided by Moss et al. (2016). We divided of the 32 odors into two lists of 16 odors (List 1 and List 2), each containing 16 odors (eight high- and eight low-familiar), ensuring that each list included odors from both sources (Experiment 2 ratings and Moss et al., 2016). The division was done to ensure that the familiarity and pleasantness ratings of the two lists did not significantly differ (see Table S4 in the Online Supplementary Material for rating comparisons; see Table S3 in the Online Supplementary Material for the composition of List 1 and List 2).

The target images used in the PA paradigm included the 32 black and white shapes used in Experiments 1 and 2, along with 32 additional abstract shapes prepared by the authors (see Fig. S1 in the Online Supplementary Materials). We added these extra shapes because the number of trials in Experiment 3 was doubled compared to Experiments 1 and 2.

#### Procedure

Participants were recruited separately for an experimental group and a control group and could only participate in one group or the other. The experimental group underwent 4 weeks of odor training at home, conducted once a week. They then participated in a laboratory session for PA training and testing. The control group only attended the laboratory session.

#### Odor-training sessions

After the volunteers were given a detailed explanation of the procedure and signed the informed consent form, they were given four packages for each week separately. Both high- and low-familiar odors were included in the training packages, with half of the experimental group receiving the packages for list 1 and the other half for list 2. Each package contained 16 aroma-sticks, dimension 11.5 × 1.5 × 0.1 cm, made of paper with soy ink and provided by Supvox company under ASIN: B082B5H1SP. To prepare the packages, the last two centimeters of each aroma stick were dipped in a scented oil and then placed in a zippered plastic bag. Each aroma stick was coded with a three-digit code (combination of letters and numbers), and each package was labeled with a week number and a QR code. By scanning the QR code, the participant gained access to online rating survey of odors for that specific week (www.soscisurvey.de).

The participant’s task was to scan the QR code or open the link for the week that was emailed to them. They then had to enter the participant code, read the instructions, pick up an aroma stick in any order, take it out of the zippered plastic bag, enter the code of the aroma stick into the corresponding box, sniff it, and rate the odors on eight ratings based on a 7-point Likert scale from not at all to very much (see Table 2 for the English version of the questions. For the German version of the questions, see Table S5 in the Online Supplementary Materials). The task was intended to simulate the complex cognitive processes involved in real-world odor familiarization, such as attentive sniffing and subjective evaluation, beyond mere exposure.

**Table 2:** Questions for each rating in the odor training phase of Experiment 3

Question type	Lower scale	Upper scale
Familiarity		
How familiar is the smell to you?	Not at all	Very
Frequency		
How often do you smell this odor?	Not at all	Very
Pleasantness		
How pleasant do you find the smell?	Not at all	Very
Irritability		
How arousing/exciting/activating do you find the smell?	Not at all	Very
Context availability		
How easily can you remember at least one situation in which you encountered the smell?	Not at all	Very
Discrimination		
How confidently can you discriminate this odor from others?	Not at all	Very
Intensity		
How intense is this smell?	Not at all	Very
Complexity		
How complex is this smell?	Not at all	Very

The average time interval between the odor training sessions was 7 days (*SD* =.75, minimum = 2, maximum = 12) and the average time interval between the last training session (i.e. session 4 of training) and laboratory session was *M* = 8.61 days (*SD* = 4.89, minimum = 3, maximum = 26^2^).

#### Laboratory session

As in Experiments 1 and 2, the laboratory session was conducted in the laboratory room with the same equipment and the same software. The experiment consisted of three phases: an encoding phase, a distraction phase, and a retrieval phase, identical to those in Experiments 1 and 2, except that the encoding phase involved eight blocks to associate 32 pairs of stimuli, and the retrieval phase also involved eight blocks to evaluate the associations with 32 odors. In the experimental group, the odors were divided into four categories: trained high-familiar, trained low-familiar, untrained high-familiar, and untrained low-familiar, with eight odors in each category. In contrast, the control group had only two categories of odors: untrained high-familiar and untrained low-familiar, each consisting of 16 odors.

### Results

#### Odor training effect on odor ratings

**Fig. 5:**
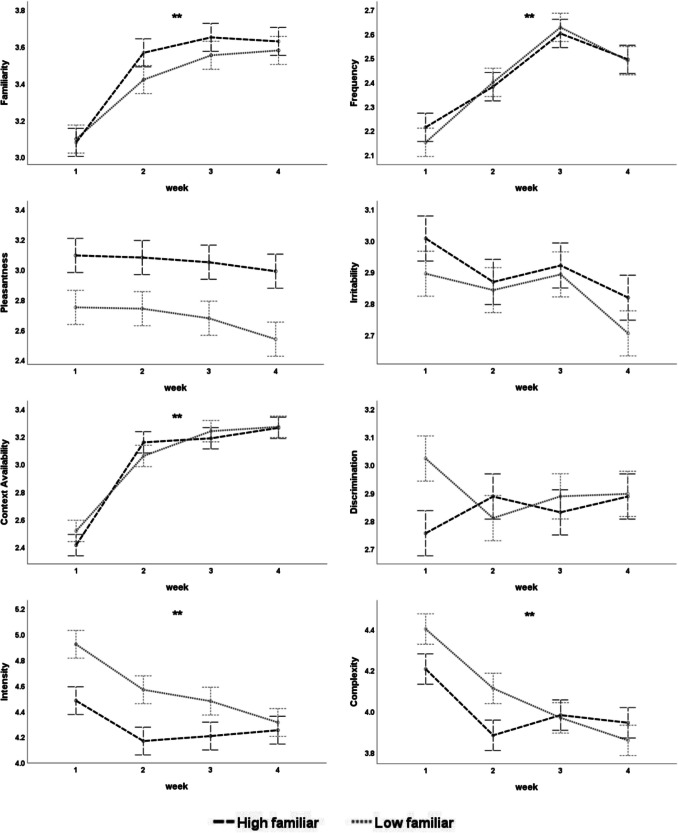
The mean rating of odor on eight ratings across the eight dimensions assessed during the odor training sessions, plotted separately for predetermined high- and low-familiar odors in Experiment 3. Error bars indicate ± 1 standard error of the mean. Significant differences in training effect (week) are indicated by asterisks (**, *p*-value <.01)

To evaluate our first hypothesis, whether odor training would change odor characteristics from the first to the fourth training week, we compared odor ratings from the first to the fourth week of training using a general linear model with IBM SPSS Statistics (version 29). Training week (weeks 1−4) and familiarity level (high vs. low) were included as fixed factors, and list of odors was included as a random factor. The results showed that familiarity, frequency, and situation availability were significantly increased for both odor categories during the training weeks. Perceptual intensity of low-familiar odors and complexity of both odor categories were significantly decreased, but pleasantness, discrimination, and irritability were not changed by odor training (see Fig. 5 and Table S6 in the Online Supplementary Materials).

#### Odor training effect on PA memory performance

To determine whether 4 weeks of odor training, conducted once a week, affects performance of high- and low-familiar odors in PA memory, we performed two sets of mixed ANOVAs comparing the experimental group (trained odors only) and the control group (no training), using Familiarity Level (high vs. low) as a within-subject factor, and Group (experimental and control) as a between-subject factor. The dependent variables were response accuracy and RT.

**Fig. 6:**
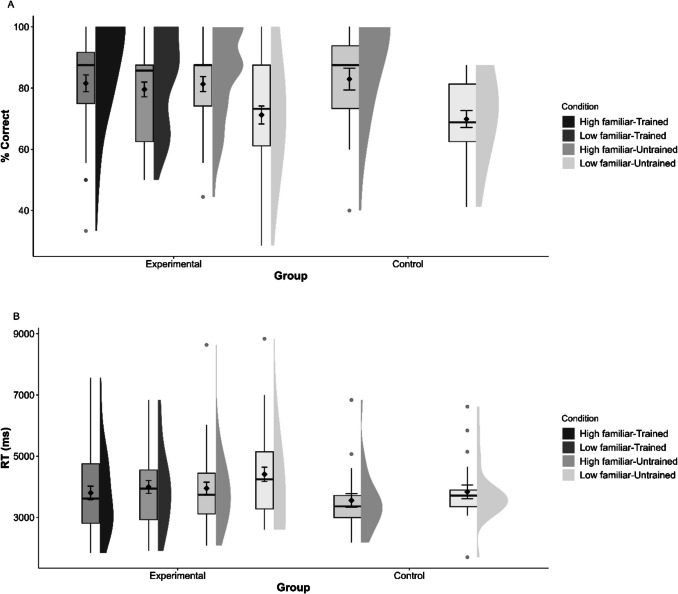
(**A**) Mean response accuracy and (**B**) reaction times (RTs) plotted separately for high- and low-familiar trained and untrained odors in the two groups in Experiment 3. The whiskers extend from the box to the smallest and largest values within 1.5 times the interquartile range from Q1 and Q3, respectively. Gray circles indicate values outside these hinges (outliers). The means (black diamond) and ± 1 standard errors are superimposed on the boxplots. Half-violins plots show distribution density for each condition

For accuracy, the ANOVA revealed a significant main effect of Familiarity Level (*F*(1, 55) = 11.17, *p* =.002, ${\eta }_{p}^{2}$ =.169). The interaction between Familiarity Level and Group was also significant (*F*(1, 55) = 6.03, *p* =.017, ${\eta }_{p}^{2}$ =.099), but the main effect of Group was not significant (*F*(1, 55) = 1.041, *p* =.241, ${\eta }_{p}^{2}$ =.025). Pairwise analyses with Bonferroni corrections indicated no significant difference between high-familiar trained odors in the experimental group and high-familiar odors in the control group (*M*_Difference_ = 1.375, *p* =.761). In contrast, low-familiar trained odors in the experimental group were recalled significantly more accurately than low-familiar odors in the control group (*M*_Difference_ = 9.687, *p* =.014) (see Fig. 6A).

For RT, the ANOVA showed a significant main effect of Familiarity Level (*F*(1, 55) = 4.04, *p* =.049, ${\eta }_{p}^{2}$ =.068), but neither the interaction between Familiarity Level and Group (*F*(1, 55) =.128 *p* =.722, ${\eta }_{p}^{2}$ =.002) nor the main effect of Group (F (1, 55) =.443, *p* =.508, ${\eta }_{p}^{2}$ =.008) was significant. Pairwise analyses with Bonferroni corrections revealed no significant differences in RTs between experimental and control groups for either high-familiar odors (*M*_Difference_ = 246 ms, *p =*.461) or low-familiar odors (*M*_Difference_ = 162 ms, *p* =.620) (see Fig. 6B).

Taken together, these findings partially support our hypothesis: odor training selectively enhanced PA memory accuracy for low-familiar odors but had no effect on RTs or on the performance for high-familiarity odors.

#### Odor training transfer effect

To evaluate our third hypothesis that the odor training effect is specific to the trained odors, with no transfer effect to untrained odors, we conducted two sets of mixed ANOVAs. First, we compared trained and untrained odors for the experimental group only on the two dependent variables: response accuracy and reaction time. Familiarity Level (high vs. low) and Training Type (trained, untrained) were entered as within-subject factors. Next, we ran another set of mixed ANOVAs, comparing the experimental group (untrained odors only) and the group control (no training), using Familiarity Level (high vs. low) as a within-subject factor, and Group (experimental and control) as a between-subject factor.^3^

For accuracy, the mixed ANOVA with the experimental group only revealed a significant interaction of Familiarity Level and Training Type (*F*(1, 35) = 4.95, *p* =.033, ${\eta }_{p}^{2}$ =.124) and a significant main effect of Familiarity Level (*F*(1, 35) = 9.98, *p* =.003, ${\eta }_{p}^{2}$ =.222). The main effect of training type was not significant (*F*(1, 35) = 2.41, *p* =.130, ${\eta }_{p}^{2}$ =.064). Pairwise analyses with Bonferroni corrections showed that for untrained odors, there was a lower accuracy of responses for low-familiar than high-familiar odors (*M*_Difference_ = 8.339, *p* =.013, Cohen’s* d* = 0.823). Additionally, low-familiar odors had a lower accuracy when untrained compared to trained (*M*_Difference_ = 10.069, *p* <.001, Cohen’s* d* = 0.434). There were no significant differences between the other three categories of odors (trained high-familiar, trained low-familiar, and untrained high-familiar) (largest *M*_Difference_ = 1.996, smallest *p* =.26) (see Fig. 6A).

For RT, the mixed ANOVA showed no significant interaction of Familiarity Type and Training Type (*F*(1, 35) = 1.66, *p* =.206, ${\eta }_{p}^{2}$ =.045) and no main effect of Training Type (*F*(1, 35) = 2.06, *p* =.161, ${\eta }_{p}^{2}$ =.055), but a significant main effect of Familiarity Level (*F*(1, 35) = 7.03, *p* =.012, ${\eta }_{p}^{2}$ =.167). Pairwise analyses with Bonferroni corrections showed that for untrained odors, RTs were slower for low-familiar than high-familiar odors (*M*_Difference_ = 459 ms, *p* =.008, Cohen’s* d* = 0.469). Additionally, low-familiar odors had a slower RTs when untrained compared to trained (*M*_Difference_ = 413 ms, *p* =.043, Cohen’s* d* = 0.321). There were no significant differences between the other three categories of odors (trained high-familiar, trained low-familiar, and untrained high-familiar) (largest *M*_Difference_ = 195 ms, smallest *p* =.11) (see Fig. 6B).

Next, we compared untrained odors in the experimental and control groups. For accuracy, the ANOVA showed a significant main effect of Familiarity Level (*F*(1, 55) = 50.04, *p* <.001, ${\eta }_{p}^{2}$ =.476). Neither the interaction between Familiarity Level and Group (*F*(1, 55) =.84, *p* =.365, ${\eta }_{p}^{2}$ =.015) nor the main effect of Group was significant (*F*(1, 55) =.001, *p* =.971, ${\eta }_{p}^{2}$ =.001). Pairwise analyses with Bonferroni corrections revealed significant differences between high- and low-familiar untrained odors in both the experimental (*M*_Difference_ = 10.07, *p* <.001) and the control group (*M*_Difference_ = 13.06, *p* <.001) (see Fig. 6A).

For RTs, the ANOVA again showed a significant main effect of Familiarity Level (*F*(1, 55) = 9.28, *p* =.004, ${\eta }_{p}^{2}$ =.144), with no significant interaction between Familiarity Level and Group (*F*(1, 55) =.55, *p* =.461, ${\eta }_{p}^{2}$ =.011) or main effect of Group (F (1, 55) = 2.404, *p* =.127, ${\eta }_{p}^{2}$ =.042). Pairwise analyses with Bonferroni corrections showed a significant difference between high- and low-familiar untrained odors in the experimental group (*M*_Difference_ = 459 ms, *p =*.003), but not in the control group (*M*_Difference_ = 279 ms, *p* =.153) (see Fig. 6B).

Overall, these results confirmed our hypothesis: the odor training effect was specific to low-familiar trained odors, with no evidence of transfer to untrained odors in either accuracy or RT performance.

### Discussion

The results of this experiment show that 4 weeks of odor training, consisting of one session per week, is sufficient to significantly increase the perceived familiarity of an odor while decreasing its intensity and complexity. These findings are consistent with previous evidence suggesting that even brief, repeated exposure can alter specific characteristics of odors, making them more familiar and less intense or complex over time (Luisier et al., 2019; Morquecho-Campos et al., 2019). The fact that pleasantness, discrimination, and irritability remained unchanged after the training aligns with previous research, indicating that the relationship between odor familiarity and pleasantness is more complex and likely non-linear (Delplanque et al., 2008). This implies that while familiarity can be enhanced through exposure, it does not necessarily lead to increased pleasantness, nor does it significantly affect the perceptual irritability of the odor.

Moreover, the findings from this study indicate that 4-week odor training can enhance the efficacy of low-familiar odors in a PA memory paradigm. Specifically, the training improved participants’ ability to form and retrieve associations involving low-familiar odors, suggesting that attentive exposures through structured odor training can significantly bolster associative memory and retrieval of targets associated with these low-familiar odors. Interestingly, the effect of training was not observed for high-familiar odors, even though their subjective familiarity increased, suggesting that simply increasing perceived familiarity does not always enhance the effectiveness of cues when the odors are already highly familiar. This finding, consistent with previous studies (Jehl et al., 1995; Olofsson et al., 2020), underscores the importance of familiarity in associative processes within olfactory memory and suggests that the benefits of odor training may be more pronounced for odors with initially weaker cognitive representations (Jehl et al., 1995; Olofsson et al., 2020).

The odor-rating task, which required participants to rate eight odor characteristics, likely engaged higher-level cognitive processes such as odor source recall or semantic evaluation. Concerns have been raised that such cognitive engagement may enhance fluent processing of stimuli, potentially affecting PA performance. We designed the evaluation task to mimic the complex cognitive mechanisms of real-world odor familiarization and hypothesized that structured engagement would enhance PA memory, particularly for unfamiliar odors. Our results support this, showing that odor training significantly improved PA performance for low-familiar trained odors compared to low-familiar untrained odors and low-familiar odors in the control group. Notably, both high- and low-familiar odors were included in the training. If cognitive processing during rating increased stimulus fluency across the board, we would expect improved PA performance for all trained odors. However, no significant training effect was observed for high-familiar odors, suggesting that the cognitive engagement that specifically facilitated PA performance was specific to low-familiar odors, which benefit more from structured training. The inclusion of untrained odors and a control group further confirmed the specificity of training effects. However, we acknowledge that explicit rating may introduce cognitive demands distinct from implicit odor processing. Future studies could explore this by comparing training with and without rating tasks to assess processing fluency directly. These findings underscore the efficacy of our training design while identifying avenues for further investigation.

However, our results also reveal that odor training did not produce a transfer effect to untrained odors, as there was no significant difference in olfactory PA performance for untrained odors between the experimental and control groups. This, in contrast with previous research (Morquecho-Campos et al., 2019; Olofsson et al., 2020), suggests that the benefits of odor training, in terms of how effective the odors are as associative cues, are odor-specific and do not generalize to other, untrained odors. This lack of transfer effect highlights the specificity of olfactory learning and suggests that the mechanisms underlying odor familiarity enhancement may be closely tied to the particular odors involved in the training. Consequently, while short-term odor training can improve memory for low-familiarity odors, its efficacy may be limited to those specific odors, without broader generalization to other olfactory stimuli.

## General discussion

The aim of this study was to examine the impact of odor familiarity on odor PA memory performance. Additionally, we explored whether increasing odor familiarity through training could improve their effectiveness as cues in the PA memory task. The findings from the three experiments presented in this study provide deeper insight into how odor familiarity affects olfactory PA memory. We have four main findings related to:

1. The impact of odor familiarity, frequency and discrimination on the odor PA memory paradigm,
2. The impact of odor training on PA memory performance,
3. Odor-specificity of odor training, and
4. How odor ratings are affected by odor training. We discuss each of these four points in more detail below.

### Odor familiarity and its effect on odor PA memory

In Experiments 1 and 2, we demonstrated that odor familiarity and frequency significantly influence odor PA memory performance. Our findings revealed that high-familiar and frequent odors improved PA performance by eliciting quicker and more accurate target recognition. This finding, consistent with previous studies (Davis, 1975, 1977), indicates that odor familiarity has a positive effect on odor associative memory. Lyman and McDaniel (1990) found that memory for familiar odors was better than memory for unfamiliar odors. Larsson and Bäckman (1997) also found that recognition performance was correlated with both rated familiarity and label quality. While perceived odor discriminability contributed to improved accuracy in some analyses, its effect was not consistently significant across experiments, suggesting a limited role in odor PA memory compared to familiarity and frequency. Overall, our results, alongside prior studies, suggest that odor PA memory is primarily strengthened by odor familiarity and frequency, with discriminability playing a less consistent role.

An important consideration in interpreting our findings is the potential role of odor naming in PA memory performance. In Experiments 1 and 2, participants provided explicit verbal labels for the odors, and we observed a strong correlation between odor familiarity and verbalizability (*r* =.872), suggesting that familiarity may partly reflect an ability to assign a name to an odor. Further analysis revealed a moderate but significant correlation between verbalizability and PA memory performance for each odor across Experiments 1 and 2 (*r* =.458, *p* =.024), raising the possibility that verbal labeling contributed to memory encoding. However, in Experiment 3, participants were not asked to rate or explicitly label the odors. The control group followed the same procedure as in Experiments 1 and 2 − minus the odor training and odor labeling − yet their PA performance closely mirrored that of earlier experiments. This focus on the control group is important because it allows us to assess memory performance in the absence of both odor training and explicit verbalization. These findings suggest that while explicit verbalizability may enhance performance, it is not a necessary condition for the observed effects of odor familiarity on memory. Nevertheless, we cannot rule out the possibility that participants engaged in implicit verbal labeling during encoding, even when not instructed to do so. Future research will be needed to disentangle the relative contributions of odor familiarity and both explicit and implicit verbalization to olfactory memory performance.

A potential limitation of our approach concerns the use of a self-reported measure of odor discriminability. Prior studies have shown that subjective ratings of olfactory discrimination do not always align with objective measures, such as those obtained from standardized tests like the Sniffin’ Sticks test battery (e.g., Adams et al., 2016; Philpott et al., 2006). This suggests that self-rated discriminability may capture perceived distinctiveness rather than objective ability. Future research should incorporate objective measures of odor discrimination ability to better evaluate the validity of self-report measures and clarify their relationship with PA memory performance and enhance the robustness of findings.

### The impact of odor training on PA memory

In Experiment 3, we found that a 4-week odor training program, conducted weekly, significantly enhanced the efficacy of low-familiar odors in a PA memory paradigm. Specifically, trained low-familiar odors showed higher accuracy and faster reaction compared to untrained low-familiar odors in the experimental group. Low-familiar odors are thought to typically have weaker memory representations due to limited prior exposure (Stevenson & Mahmut, 2013), leading to lower accuracy and slower retrieval in memory tasks. Our results suggest that odor training, through repeated active exposure (e.g., odor-rating tasks), strengthens their cognitive and perceptual representation, thereby improving their effectiveness as cues in PA tasks.

Our findings highlight the potential for odor training to enhance memory performance, particularly for low-familiar odors, but also reveal areas for further exploration. Future studies should investigate how these mechanisms like sensitivity and discrimination translate to improved encoding and retrieval and explore the contributions of semantic and emotional associations to these processes. Moreover, longitudinal studies could examine how sustained odor training impacts memory over longer periods and whether similar effects occur in real-world contexts.

While odor training significantly enhanced memory performance for low-familiar odors, its effect was less pronounced for high-familiar odors. This may be because these odors already have strong and distinct memory representations due to frequent encounters. Future research should examine whether training can enhance memory performance for high-familiar odors in more complex or demanding tasks, shedding light on the limits of the impact of familiarity on odor memory.

Finally, a methodological limitation of the present study is the absence of an active control group that engaged in a comparable, non-odor task, such as sniffing unscented stimuli. Although our control group was designed to establish a baseline for memory performance without training exposure, the absence of a task-matched control group leaves open the possibility that differences in task engagement or expectancy contributed to the observed group differences. Although implementing such a control presents practical and conceptual challenges − including concerns about participant compliance, environmental contamination, and demand characteristics − it would be valuable in future studies to include such a group to better isolate the effects of odor-specific training from more general task-related influences.

### Odor training is odor-specific

The results of Experiment 3 provide strong evidence that the benefits of odor training are highly odor-specific and do not generalize to other, untrained odors. This finding aligns with previous research (Rabin, 1988; Tempere et al., 2012), indicating that olfactory learning is often stimulus-bound, with improvements in recognition or discrimination tied closely to the specific odors used during training. The lack of generalization to novel, untrained odors suggests that the mechanisms underlying this learning, such as enhanced familiarity for the trained odorants.

In contrast to other sensory modalities, where generalization is more commonly observed (e.g., in touch: Harrar et al., 2014; in vision: Manenti et al., 2023; and in audition: Wright et al., 2010), olfactory learning seems to result in highly targeted perceptual improvements, which are not easily extended to other, similar stimuli (Oleszkiewicz et al., 2021). The odor-specific nature of odor training may be attributed to the individualized and complex nature of odor perception, where the brain’s representation of an odor is shaped not only by its physical properties but also by personal experience and prior knowledge (Gottfried, 2008; Li et al., 2008). Consequently, while the training increases familiarity with specific odors, it does not enhance sensitivity to untrained ones, underscoring the specificity of olfactory learning and the limited transfer of learning effects across different odors.

### Odor ratings are affected by odor training

The findings from Experiment 3 provide valuable insights into the effects of odor training on ratings of odors. The significant increase in perceived familiarity after just four weeks of training highlights the potency of repeated exposure in shaping our sensory experience. These results align with prior research (Luisier et al., 2019; Morquecho-Campos et al., 2019), where odor training has been shown to alter specific perceptual characteristics of odors.

Interestingly, as familiarity increased, the perceived intensity and complexity of the odors decreased. This suggests that as individuals become more accustomed to an odor, they may perceive it as less overwhelming or intricate.

These results suggest that while odor training can alter certain dimensions of odor perception − namely familiarity, intensity, and complexity − it may not be sufficient to change other aspects like pleasantness, irritability, or discrimination. This highlights the selective nature of how training impacts sensory perception and underscores the need for more targeted approaches if the goal is to modify specific perceptual qualities beyond familiarity.

## Conclusion

The findings of this study shed light on the intricate relationship between odor familiarity and odor PA memory performance. Consistent with previous studies (Davis, 1975, 1977), we observed that high-familiar odors led to more accurate and faster target recognition compared to low-familiar odors. Importantly, our study goes further by investigating the effect of odor training on odor PA memory performance. The results reveal that 4-week odor training improves the efficacy of low-familiar odors in a PA memory paradigm but does not offer additional benefits for high-familiar odors.

The reported findings may have broad applications, suggesting that odor training could serve as a cognitive tool to enhance memory and learning in various contexts, including clinical settings, educational environments, or forensic psychology. The findings underscore the plasticity of the olfactory system and its capacity for training-induced change, opening avenues for future research into odor-based interventions.

## Supplementary Information

Below is the link to the electronic supplementary material.

Supplementary file1 (DOCX 214 KB)

## Data Availability

The materials, programs, and data that support the findings reported in this paper are openly available on the Open Science Framework at: https://osf.io/myqrw/

## Footnotes

1 Although the sample size was determined a priori to ensure sufficient statistical power, visual inspection of the accuracy data (see Fig. 3) suggested deviations from normality and the presence of a potential outlier in the high-familiarity condition. To ensure the robustness of the results, we performed a nonparametric Wilcoxon signed-rank test as a supplementary analysis. The result of the Wilcoxon test confirmed the result of the paired samples t-test, with a statistically significant difference between the high- and low-familiarity conditions, *Z* = 40.00, *p* =.013. This suggests that the original result is robust to violations of the normality assumption.

2 One subject was identified as an outlier with regard to the time interval between the last training session and the laboratory session. The maximum time interval without the outlier was 18. However, we kept this subject in the analysis because excluding them did not change the overall pattern of results.

3 Because of the unequal group numbers, we performed Levene’s test of equality of error variances in SPSS for the two dependent variables to ensure that the assumption of homogeneity of variances was met. The results confirmed that this assumption was met, allowing us to proceed with the ANOVA analysis.

## Code availability

The R code that has been used to plot the statistical figures is available in the same repository as above.
