# Supplementary material for: Familiarity enhances the effectiveness of odors as cues in paired-associate memory
Source: Mem Cognit. 2025 Sep 3;54(3):959–79. doi: 10.3758/s13421-025-01790-1 (PMC13133209; doi:10.3758/s13421-025-01790-1)
Supplement: Supplementary file 1 — Supplementary file1 (DOCX 214 KB) [file 13421_2025_1790_MOESM1_ESM.docx]

**Odor Familiarity Enhances Odor Paired Associate Memory performance**

Mohammad Hamzeloo, Luisa Bogenschüz^,^ Ryan P. M. Hackländer, and Christina Bermeitinger

University of Hildesheim

**Supplementary Materials**

**Experiment 1**

**Table S1.**

List of odors used in Experiments 1 and 2. Note that the odor condition in Experiment 2 was based on individual ratings, and for any particular subject does not align with the odor condition listed in the table.

|  | **Odor** | **Familiarity type** | **Familiarity** | **Pleasantness** |
| --- | --- | --- | --- | --- |
| 1 | Marzipan | High familiar | 6.12 | 4.96 |
| 2 | Aniseed Balls | High familiar | 5.88 | 3.98 |
| 3 | Sports Rub | High familiar | 5.60 | 4.21 |
| 4 | Baby powder | High familiar | 5.48 | 5.46 |
| 5 | Tea Tree oil | High familiar | 5.20 | 3.67 |
| 6 | Garlic | High familiar | 5.10 | 2.06 |
| 7 | Hospital Modern Day | High familiar | 5.06 | 4.32 |
| 8 | Sage | High familiar | 4.96 | 4.12 |
| 9 | Soap Suds | High familiar | 4.90 | 4.37 |
| 10 | Washday | High familiar | 4.78 | 3.96 |
| 11 | Earthy | High familiar | 4.76 | 3.80 |
| 12 | Chocolate | High familiar | 4.67 | 3.80 |
| 13 | Onion | High familiar | 4.66 | 2.00 |
| 14 | Out at Sea | High familiar | 4.65 | 3.67 |
| 15 | Rosemary | High familiar | 4.53 | 3.84 |
| 16 | Hazelnut | High familiar | 4.40 | 3.25 |
| 17 | Gingerbread | Low familiar | 3.69 | 3.51 |
| 18 | Cedar | Low familiar | 3.68 | 3.36 |
| 19 | Vanilla | Low familiar | 3.66 | 3.76 |
| 20 | Rockpools | Low familiar | 3.61 | 4.04 |
| 21 | Leather/Hide | Low familiar | 3.60 | 3.55 |
| 22 | Candy Floss | Low familiar | 3.43 | 3.65 |
| 23 | Peat | Low familiar | 3.31 | 3.94 |
| 24 | [Boiler Room](https://aromaprime.com/boiler-room-aroma-oil/) | Low familiar | 3.04 | 2.42 |
| 25 | Malted Barley | Low familiar | 3.20 | 3.70 |
| 26 | Nag Champa | Low familiar | 3.10 | 3.73 |
| 27 | Clove Oil | Low familiar | 3.04 | 2.58 |
| 28 | Cinder Toffee | Low familiar | 3.02 | 3.59 |
| 29 | Pencils | Low familiar | 3.02 | 3.33 |
| 30 | Wood Chip | Low familiar | 2.94 | 3.80 |
| 31 | Sandalwood | Low familiar | 2.73 | 3.73 |
| 32 | Buttered Popcorn | Low familiar | 2.72 | 3.02 |

The odor conditions differed significantly in their familiarity (t_31_ = 12.09, *p* < 0.001), but not in their pleasantness ratings (t_31_ = 1.45, *p* = 0.16).

**Table S2.**

Rating questions for each dimension in the rating phase in German language.

| Question type | Lower scale | Upper scale |
| --- | --- | --- |
| Familiarity |  |  |
| Wie vertraut ist Dir der Geruch? | Gar nicht | Sehr vertraut |
| Wie bekannt ist Dir der Geruch? | Gar nicht | Sehr vertraut |
| Wie sicher bist Du Dir, dass du diesen Geruch bereits gerochen hast? | Gar nicht | Sehr sicher |
| Frequency |  |  |
| Wie oft hast Du den Geruch in Deinem Leben bereits gerochen? | Gar nicht | Sehr oft |
| Wie oft hast Du den Geruch in den letzten 12 Monaten gerochen? | Gar nicht | Sehr oft |
| Wie oft hast Du den Geruch in den letzten 4 Wochen gerochen? | Gar nicht | Sehr oft |
| Pleasantness |  |  |
| Wie angenehm findest Du den Geruch? | Sehr unangenehm | Sehr angenehm |
| Irritability |  |  |
| Wie erregend/ aufregend/ aktivierend findest Du den Geruch? | Gar nicht | Sehr |
| Context availability |  |  |
| Wie leicht kannst du dich an mindestens eine Situation erinnern, in der dir dieser Geruch begegnet ist? | Gar nicht | Sehr |
| Discrimination |  |  |
| Wie sicher bist du, dass du diesen Geruch von anderen Gerüchen unterscheiden kannst? | Gar nicht | Sehr sicher |
| Age of acquisition |  | |
| In welchem Alter hast du Sie diesen Geruch zum ersten Mal wahrgenommen? | Open-ended question | |
| Verbalizability |  | |
| Welcher Geruch ist das? Bitte geben Sie, wenn möglich, eine verbale Bezeichnung für diesen Geruch ein. | Open-ended question | |

**Experiment 3**

**Table S3.**

List of odors used in Experiment 3.

|  | **Odor** | **List*** | **Familiarity type** | **Familiarity** | **Pleasantness** |
| --- | --- | --- | --- | --- | --- |
| 1 | Marzipan | 1 | High familiar | 6.10 | 4.85 |
| 2 | Peach | 1 | High familiar | 5.82 | 4.40 |
| 3 | Baby powder | 1 | High familiar | 5.68 | 5.08 |
| 4 | Aniseed Balls | 1 | High familiar | 5.64 | 3.75 |
| 5 | Banana | 1 | High familiar | 5.40 | 5.32 |
| 6 | Tea tree oil | 1 | High familiar | 5.20 | 3.67 |
| 7 | Sage | 1 | High familiar | 5.14 | 4.45 |
| 8 | Aftershave | 1 | High familiar | 4.96 | 4.74 |
| 9 | Beef | 1 | Low familiar | 3.80 | 1.88 |
| 10 | Boiler Room | 1 | Low familiar | 3.71 | 2.69 |
| 11 | Barbecue | 1 | Low familiar | 3.63 | 2.30 |
| 12 | Wood Chip | 1 | Low familiar | 3.67 | 3.59 |
| 13 | Blue Cheese | 1 | Low familiar | 3.51 | 1.89 |
| 14 | Ginger Bread | 1 | Low familiar | 3.48 | 3.36 |
| 15 | Cinder Toffee | 1 | Low familiar | 3.02 | 3.59 |
| 16 | Sandalwood | 1 | Low familiar | 3.01 | 3.24 |
| 17 | Sports Rub | 2 | High familiar | 5.60 | 4.21 |
| 18 | Bubble Gum | 2 | High familiar | 5.53 | 5.41 |
| 19 | Soap Suds | 2 | High familiar | 5.53 | 4.59 |
| 20 | Out at Sea | 2 | High familiar | 5.27 | 4.74 |
| 21 | Vanilla | 2 | High familiar | 5.26 | 5.41 |
| 22 | Mulled Wine | 2 | High familiar | 5.08 | 5.14 |
| 23 | Malted Barley | 2 | High familiar | 4.94 | 4.82 |
| 24 | Wash day | 2 | High familiar | 4.78 | 3.96 |
| 25 | Whisky | 2 | Low familiar | 3.61 | 2.59 |
| 26 | Cedar | 2 | Low familiar | 3.65 | 3.36 |
| 27 | Carbolic Soap | 2 | Low familiar | 3.61 | 3.12 |
| 28 | Rockpools | 2 | Low familiar | 3.61 | 4.04 |
| 29 | Pencils | 2 | Low familiar | 3.57 | 3.66 |
| 30 | Coal pit | 2 | Low familiar | 3.49 | 1.67 |
| 31 | Myrrh | 2 | Low familiar | 3.71 | 3.86 |
| 32 | Peat | 2 | Low familiar | 3.41 | 2.49 |

*List 1 and List 2 are balanced lists comprising odors from both Experiment 2 ratings and Moss et al. (2016).

**Table S4:** Results of *t*-tests for familiarity and pleasantness in the two lists (assuming equal variances).

|  | List 1  Familiarity | List 2  Familiarity | List 1  Pleasantness | List 1  Pleasantness |
| --- | --- | --- | --- | --- |
| Mean | 4.485235 | 4.415064 | 3.673927 | 3.941409 |
| Variance | 1.195424 | 0.785714 | 1.192756 | 1.188737 |
| df | 30 |  | 30 |  |
| *t* | .199416 |  | -.69331 |  |
| *P*(two-tail) | .843283 |  | .493446 |  |

**Figure S1.**

The additional target stimuli used in Experiment 3.

| 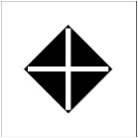 | 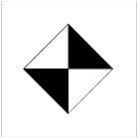 | 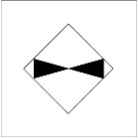 | 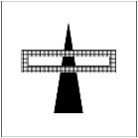 | 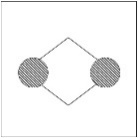 | 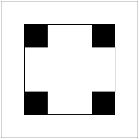 | 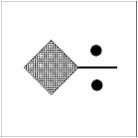 | 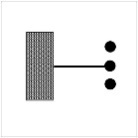 |
| --- | --- | --- | --- | --- | --- | --- | --- |
| 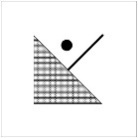 | 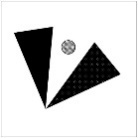 | 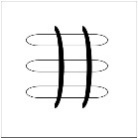 | 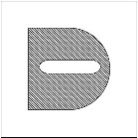 | 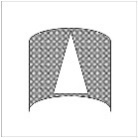 | 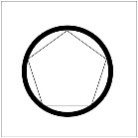 | 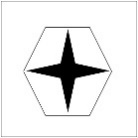 | 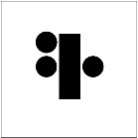 |
| 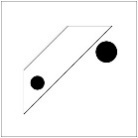 | 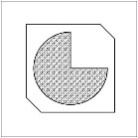 | 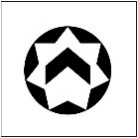 | 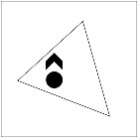 | 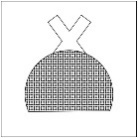 | 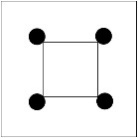 | 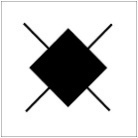 | 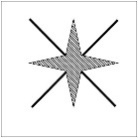 |
| 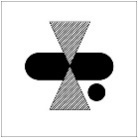 | 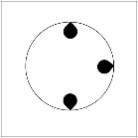 | 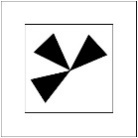 | 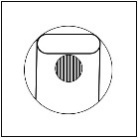 | 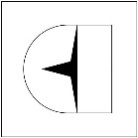 | 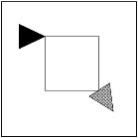 | 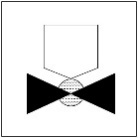 | 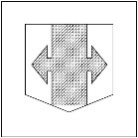 |

**Table S5.**

Rating questions for each dimension in familiarization training in Experiment 3 in German language.

| Question type | Lower scale | Upper scale |
| --- | --- | --- |
| Familiarity |  |  |
| Wie bekannt ist Dir der Geruch? | Gar nicht | Sehr |
| Frequency |  |  |
| Wie häufig begegnen Sie diesem Geruch? | Gar nicht | Sehr |
| Pleasantness |  |  |
| Wie angenehm findest Du den Geruch? | Gar nicht | Sehr |
| Irritability |  |  |
| Wie erregend/ aufregend/ aktivierend findest Du den Geruch? | Gar nicht | Sehr |
| Context availability |  |  |
| Wie leicht können Sie sich an mindestens eine Situation erinnern, in der Ihnen der Geruch begegnet ist? | Gar nicht | Sehr |
| Discrimination |  |  |
| Wie sicher sind Sie, dass Sie diesen Geruch von anderen unterscheiden können? | Gar nicht | Sehr |
| Intensity |  | |
| Wie intensiv ist dieser Geruch? | Gar nicht | Sehr |
| Complexity |  | |
| Wie komplex ist dieser Geruch? | Gar nicht | Sehr |

**Table S6**

Odor perceptual rating results separated by each week of familiarization training in Experiment 3.

|  | Familiarity type^b^ | Week 1 | | Week 2 | | Week 3 | | Week 4 | | *p* value^f^ | | |
| --- | --- | --- | --- | --- | --- | --- | --- | --- | --- | --- | --- | --- |
| Odor evaluation^a^ |  | M | SD | M | SD | M | SD | M | SD | Week^c^ | familiarity type^d^ | Interaction^e^ |
| Familiarity | High | 3.08 | 0.26 | 3.57 | 0.35 | 3.65 | 0.30 | 3.63 | 0.27 | <.001 | 0.197 | 0.723 |
|  | Low | 3.10 | 0.23 | 3.42 | 0.36 | 3.55 | 0.29 | 3.58 | 0.30 |  |  |  |
| Pleasantness | High | 3.09 | 0.46 | 3.08 | 0.51 | 3.05 | 0.55 | 2.99 | 0.55 | 0.551 | <.001 | 0.09 |
|  | Low | 2.75 | 0.47 | 2.74 | 0.55 | 2.68 | 0.43 | 2.54 | 0.32 |  |  |  |
| Irritability | High | 3.01 | 0.25 | 2.87 | 0.35 | 2.92 | 0.28 | 2.82 | 0.47 | 0.112 | 0.221 | 0.904 |
|  | Low | 2.89 | 0.35 | 2.84 | 0.29 | 2.89 | 0.27 | 2.70 | 0.25 |  |  |  |
| Context Availability | High | 2.41 | 0.37 | 3.16 | 0.43 | 3.19 | 0.35 | 3.26 | 0.35 | <.001 | 0.809 | 0.727 |
|  | Low | 2.52 | 0.23 | 3.06 | 0.34 | 3.24 | 0.50 | 3.27 | 0.30 |  |  |  |
| Discrimination | High | 2.75 | 0.48 | 2.89 | 0.45 | 2.83 | 0.47 | 2.89 | 0.36 | 0.969 | 0.395 | 0.421 |
|  | Low | 3.02 | 0.45 | 2.81 | 0.28 | 2.89 | 0.47 | 2.90 | 0.40 |  |  |  |
| Intensity | High | 4.48 | 0.48 | 4.17 | 0.44 | 4.21 | 0.42 | 4.25 | 0.25 | <.001 | <.001 | 0.305 |
|  | Low | 4.92 | 0.45 | 4.57 | 0.57 | 4.48 | 0.44 | 4.31 | 0.37 |  |  |  |
| Complexity | High | 4.21 | 0.33 | 3.88 | 0.37 | 3.98 | 0.23 | 3.94 | 0.34 | <.001 | 0.14 | 0.117 |
|  | Low | 4.40 | 0.30 | 4.11 | 0.32 | 3.97 | 0.29 | 3.86 | 0.26 |  |  |  |
| Frequency | High | 2.21 | 0.22 | 2.38 | 0.33 | 2.60 | 0.34 | 2.49 | 0.33 | <.001 | 0.907 | 0.933 |
|  | Low | 2.15 | 0.21 | 2.40 | 0.26 | 2.63 | 0.36 | 2.49 | 0.26 |  |  |  |

^a^Ratings performed on 7-point scale

^b^High = high familiar odors, low = low familiar odors

^c^The main effect of week

^d^The main effect of odor type

^e^The interaction between week and odor type

^f^The results of a 2x4 repeated measures ANOVA
